# Supplementary material for: The role of oxytocin in delay of gratification and flexibility in non-social decision making
Source: eLife. 2021 Apr 6;10:e61844. doi: 10.7554/eLife.61844 (PMC8024008; doi:10.7554/eLife.61844)
Supplement: Supplementary file 1. [file elife-61844-supp1.docx]

**Supplementary material**

**for**

**“The role of oxytocin in delay of gratification and flexibility in non-social decision making”**

**Supplementary File 1a.** Regression coefficients for the mixed-effects regression model for the intertemporal decision task. Choices in the intertemporal decision task were regressed on fixed-effect predictors for Substance, Delay, Amount of SS reward, WMC, Order of substance administration, and the interaction terms. As participant-specific random effects, the model included predictors for Substance, Delay, Amount of SS reward, and the interaction terms.

| ***Predictors*** | ***Beta weight*** | ***Std. Error*** | ***z*** | ***p*** | ***Cohen’s d*** |
| --- | --- | --- | --- | --- | --- |
| Intercept | 6.51 | 1.96 | 3.33 | **0.001** | **0.47** |
| Substance | -0.82 | 1.09 | -0.75 | 0.454 | 0.10 |
| Order | 2.79 | 1.82 | 1.53 | 0.125 | 0.21 |
| Delay | -0.11 | 1.68 | -0.07 | 0.947 | 0.01 |
| SS reward | -7.56 | 0.97 | -7.77 | **<0.001** | **1.11** |
| WMC | 3.52 | 1.85 | 1.90 | 0.057 | 0.27 |
| Substance X Order | -2.13 | 0.79 | -2.68 | **0.007** | **0.38** |
| Substance X Delay | -0.36 | 1.12 | -0.32 | 0.750 | 0.04 |
| Order X Delay | 2.34 | 1.56 | 1.51 | 0.132 | 0.21 |
| Substance X SS reward | 1.08 | 0.93 | 1.16 | 0.244 | 0.16 |
| Order X SS reward | -0.69 | 0.78 | -0.88 | 0.379 | 0.12 |
| Delay X SS reward | -4.57 | 0.97 | -4.72 | **<0.001** | **0.67** |
| Substance X WMC | -0.74 | 0.85 | -0.88 | 0.381 | 0.12 |
| Order X WMC | -1.14 | 1.80 | -0.63 | 0.527 | 0.09 |
| Delay X WMC | 2.79 | 1.59 | 1.75 | 0.079 | 0.25 |
| SS reward X WMC | -0.87 | 0.78 | -1.12 | 0.265 | 0.15 |
| Substance X Order X Delay | -1.97 | 0.87 | -2.27 | **0.022** | **0.32** |
| Substance X Order X SS reward | 0.26 | 0.53 | 0.48 | 0.629 | 0.06 |
| Substance X Delay X SS reward | 1.31 | 0.98 | 1.34 | 0.180 | 0.19 |
| Order X Delay X SS reward | -0.08 | 0.74 | -0.11 | 0.911 | 0.01 |
| Substance X Order X WMC | -0.26 | 0.74 | -0.35 | 0.725 | 0.05 |
| Substance X Delay X WMC | -1.02 | 0.95 | -1.08 | 0.281 | 0.15 |
| Order X Delay X WMC | -1.68 | 1.53 | -1.09 | 0.274 | 0.15 |
| Substance X SS reward X WMC | -0.31 | 0.52 | -0.60 | 0.548 | 0.08 |
| Order X SS reward X WMC | -0.07 | 0.75 | -0.10 | 0.924 | 0.01 |
| Delay X SS reward X WMC | -0.96 | 0.74 | -1.29 | 0.197 | 0.18 |
| Substance X Order X Delay X SS reward | -0.21 | 0.59 | -0.35 | 0.726 | 0.05 |
| Substance X Order X D X WMC | 0.32 | 0.83 | 0.39 | 0.700 | 0.05 |
| Substance X Order X SS reward X WMC | 0.33 | 0.47 | 0.70 | 0.484 | 0.10 |
| Substance X Delay X SS reward X WMC | 0.05 | 0.59 | 0.08 | 0.934 | 0.01 |
| Order X Delay X SS reward X WMC | 0.03 | 0.70 | 0.05 | 0.961 | 0.01 |
| Substance X Order X Delay X SS reward X WMC | 0.23 | 0.51 | 0.45 | 0.653 | 0.06 |

**Supplementary File 1b.** Regression coefficients for the mixed-effects regression model for the intertemporal decision task (session 1). Choices in the first session of the intertemporal decision task were regressed on fixed-effect predictors for Substance, Delay, Amount of SS reward, and the interaction terms. The model also included predictors for Delay, Amount of SS reward, and the interaction terms as participant-specific random effects.

| ***Predictors*** | ***Beta weight*** | ***Std. Error*** | ***z*** | ***p*** | ***Cohen’s d*** |
| --- | --- | --- | --- | --- | --- |
| Intercept | 1.61 | 2.01 | 0.80 | 0.422 | 0.11 |
| Substance | 5.47 | 2.81 | 1.95 | 0.052 | 0.27 |
| Delay | -4.10 | 1.79 | -2.28 | **0.022** | **0.32** |
| SS reward | -5.99 | 0.96 | -6.27 | **<0.001** | **0.89** |
| Substance X Delay | 5.24 | 2.49 | 2.11 | **0.034** | **0.30** |
| Substance X SS reward | -1.50 | 1.31 | -1.15 | 0.248 | 0.16 |
| Delay X SS reward | -3.50 | 0.84 | -4.19 | **<0.001** | **0.59** |
| Substance X Delay X SS reward | -1.02 | 1.11 | -0.92 | 0.359 | 0.13 |

**Supplementary File 1c.** Regression coefficients for the mixed-effects regression model for the reversal learning task. Mean correct predictions after reversal in the reversal learning task were regressed on fixed-effect predictors for Substance, Previous reward, WMC, Order of substance administration, and the interaction terms. The model also included participant-specific random intercepts.

| ***Predictors*** | ***Beta weight*** | ***Std. Error*** | ***t*** | ***df*** | ***p*** | ***Cohen’s d*** |  |
| --- | --- | --- | --- | --- | --- | --- | --- |
| Intercept | 0.89 | 0.02 | 47.84 | 83.29 | **<0.001** | **6.83** |  |
| Substance | 0.03 | 0.02 | 1.90 | 134.32 | 0.058 | 0.27 |  |
| Previous reward | -0.00 | 0.01 | -0.16 | 134.43 | 0.871 | 0.02 |  |
| WMC | 0.04 | 0.02 | 1.96 | 83.29 | 0.053 | 0.27 |  |
| Order | -0.03 | 0.02 | -1.76 | 83.29 | 0.082 | 0.25 |  |
| Substance X Previous reward | -0.01 | 0.02 | -0.63 | 134.32 | 0.532 | 0.08 |  |
| Substance X WMC | -0.04 | 0.02 | -2.26 | 134.32 | **0.025** | **0.32** |  |
| Previous reward X WMC | -0.02 | 0.01 | -1.29 | 134.43 | 0.198 | 0.18 |  |
| Substance X Order | 0.04 | 0.02 | 2.49 | 134.32 | **0.013** | **0.35** |  |
| Previous reward X Order | 0.00 | 0.01 | 0.18 | 134.43 | 0.854 | 0.02 |  |
| WMC X Order | 0.02 | 0.02 | 1.03 | 83.29 | 0.305 | 0.14 |  |
| Substance X Previous reward X WMC | 0.03 | 0.02 | 1.52 | 134.32 | 0.131 | 0.21 |  |
| Substance X Previous reward X Order | -0.01 | 0.02 | -0.44 | 134.32 | 0.658 | 0.06 |  |
| Substance X WMC X Order | -0.02 | 0.02 | -1.03 | 134.32 | 0.303 | 0.14 |  |
| Previous reward X WMC X Order | -0.01 | 0.01 | -0.45 | 134.43 | 0.652 | 0.06 |  |
| Substance X Previous reward X WMC X Order | 0.00 | 0.02 | 0.16 | 134.32 | 0.871 | 0.02 |  |

**Supplementary File 1d.** Regression coefficients for the mixed-effects regression model for the reversal learning task (low WMC group). Mean correct predictions after reversal of the low WMC group in the reversal learning task were regressed on fixed-effect predictors for Substance, Previous reward, Order of substance administration, and the interaction terms. The model also included participant-specific random intercepts.

| ***Predictors*** | ***Beta weight*** | ***Std. Error*** | ***t*** | ***df*** | ***P*** | ***Cohen’s d*** |
| --- | --- | --- | --- | --- | --- | --- |
| Intercept | 0.85 | 0.03 | 28.05 | 37.83 | **<0.001** | **5.72** |
| Substance | 0.08 | 0.03 | 2.69 | 66.00 | **0.008** | **0.55** |
| Previous reward | 0.01 | 0.02 | 0.73 | 66.00 | 0.466 | 0.14 |
| Order | -0.05 | 0.03 | -1.70 | 37.83 | 0.096 | 0.34 |
| Substance X Previous reward | -0.04 | 0.03 | -1.38 | 66.00 | 0.170 | 0.28 |
| Substance X Order | 0.06 | 0.03 | 2.28 | 66.00 | **0.025** | **0.46** |
| Previous reward X Order | 0.01 | 0.02 | 0.41 | 66.00 | 0.681 | 0.08 |
| Substance X Previous reward X Order | -0.01 | 0.03 | -0.39 | 66.00 | 0.696 | 0.07 |

**Supplementary File 1e.** Regression coefficients for the mixed-effects regression model for the reversal learning task (high WMC group). Mean correct predictions after reversal of the low WMC group in the reversal learning task were regressed on fixed-effect predictors for Substance, Previous reward, Order of substance administration, and the interaction terms. The model also included participant-specific random intercepts.

| ***Predictors*** | ***Beta weight*** | ***Std. Error*** | ***t*** | ***df*** | ***p*** | ***Cohen’s d*** |
| --- | --- | --- | --- | --- | --- | --- |
| Intercept | 0.93 | 0.02 | 42.69 | 48.55 | **<0.001** | **8.53** |
| Substance | -0.00 | 0.02 | -0.27 | 68.39 | 0.782 | 0.05 |
| Previous reward | -0.02 | 0.01 | -1.13 | 68.53 | 0.259 | 0.22 |
| Order | -0.01 | 0.02 | -0.62 | 48.55 | 0.535 | 0.12 |
| Substance X Previous reward | 0.01 | 0.02 | 0.70 | 68.39 | 0.486 | 0.14 |
| Substance X Order | 0.02 | 0.02 | 1.15 | 68.39 | 0.254 | 0.23 |
| Previous reward X Order | -0.00 | 0.01 | -0.20 | 68.53 | 0.836 | 0.04 |
| Substance X Previous reward X Order | -0.00 | 0.02 | -0.22 | 68.39 | 0.826 | 0.04 |

**Supplementary File 1f.** Regression coefficients for the mixed-effects regression model for the dictator game. Choices in the dictator game were regressed on fixed-effect predictors for Substance, Inequity amount, Inequity type, WMC, Order of substance administration, and the interaction terms, controlling for Efficiency. The model also included predictors for Substance, Inequity amount, Inequity type, the interaction terms and Efficiency as participant-specific random effects.

| ***Predictors*** | ***Beta weight*** | ***Std. Error*** | ***z*** | ***p*** | ***Cohen’s d*** |
| --- | --- | --- | --- | --- | --- |
| Intercept | 29.96 | 4.34 | 6.90 | **<0.001** | 0.98 |
| Substance | -2.14 | 1.35 | -1.58 | 0.111 | 0.22 |
| Order | -0.12 | 1.25 | -0.10 | 0.919 | 0.01 |
| Inequity amount | 5.32 | 1.40 | 3.81 | **<0.001** | 0.54 |
| WMC | 0.98 | 1.31 | 0.75 | 0.451 | 0.10 |
| Inequity type | -23.09 | 3.71 | -6.22 | **<0.001** | 0.88 |
| Efficiency | 20.97 | 2.70 | 7.75 | **<0.001** | 1.10 |
| Substance X Order | -1.64 | 1.01 | -1.63 | 0.101 | 0.23 |
| Substance X Inequity amount | -2.37 | 1.23 | -1.93 | **0.052^†^** | 0.27 |
| Order X Inequity amount | 0.51 | 0.84 | 0.61 | 0.540 | 0.08 |
| Substance X WMC | -0.10 | 1.03 | -0.10 | 0.918 | 0.01 |
| Order X WMC | 2.68 | 1.28 | 2.08 | **0.036** | 0.29 |
| Inequity X WMC | 0.98 | 0.89 | 1.10 | 0.270 | 0.15 |
| Substance X Inequity type | 2.50 | 1.51 | 1.66 | 0.096 | 0.23 |
| Order X Inequity type | 0.72 | 1.59 | 0.45 | 0.648 | 0.06 |
| Inequity amount X Inequity type | -14.05 | 2.47 | -5.68 | **<0.001** | 0.81 |
| WMC X Inequity type | -0.21 | 1.65 | -0.13 | 0.896 | 0.01 |
| Substance X Order X Inequity amount | -1.12 | 0.79 | -1.43 | 0.150 | 0.20 |
| Substance X Order X WMC | 0.63 | 1.02 | 0.61 | 0.538 | 0.08 |
| Substance X Inequity amount X WMC | -0.13 | 0.79 | -0.17 | 0.863 | 0.02 |
| Order X Inequity amount X WMC | 1.85 | 0.87 | 2.13 | **0.032** | 0.30 |
| Substance X Order X Inequity type | 1.12 | 1.12 | 1.00 | 0.314 | 0.14 |
| Substance X Inequity amount X Inequity type | 3.15 | 1.43 | 2.20 | **0.027** | 0.31 |
| Order X Inequity amount X Inequity type | -0.37 | 1.13 | -0.32 | 0.742 | 0.04 |
| Substance X WMC X Inequity type | 1.19 | 1.16 | 1.03 | 0.302 | 0.14 |
| Order X WMC X Inequity type | -2.89 | 1.62 | -1.79 | 0.072 | 0.25 |
| Inequity amount X WMC X Inequity type | -1.02 | 1.18 | -0.86 | 0.385 | 0.12 |
| Substance X Order X Inequity amount X WMC | 0.29 | 0.78 | 0.37 | 0.711 | 0.05 |
| Substance X Order X Inequity amount X Inequity type | 1.43 | 0.96 | 1.50 | 0.133 | 0.21 |
| Substance X Order X WMC X Inequity type | -0.60 | 1.14 | -0.53 | 0.595 | 0.07 |
| Substance X Inequity amount X WMC X Inequity type | 0.53 | 0.96 | 0.55 | 0.575 | 0.07 |
| Order X Inequity amount X WMC X Inequity type | -2.24 | 1.15 | -1.94 | 0.051 | 0.27 |
| Substance X Order X Inequity amount X WMC X Inequity type | 0.32 | 0.95 | 0.34 | 0.731 | 0.04 |
| *† p* = 0.026 one-tailed as preregistered | | | | |  |

**Supplementary File 1g.** Regression coefficients for the mixed-effects regression model for the digit span backward task. Mean correct predictions in the digit span backward task were regressed on fixed-effect predictors for Substance, Assessment time (pretest versus posttest), Order of substance administration and their interactions. The model included also participant-specific random intercepts.

| ***Predictors*** | ***Beta weight*** | ***Std. Error*** | ***t*** | ***df*** | ***p*** | ***Cohen’s d*** |
| --- | --- | --- | --- | --- | --- | --- |
| Intercept | 0.54 | 0.03 | 20.87 | 100.31 | **<0.001** | 2.98 |
| Substance | -0.00 | 0.02 | -0.12 | 147.00 | 0.902 | 0.01 |
| Order | 0.07 | 0.03 | 2.70 | 100.31 | **0.008** | 0.38 |
| Assessment time | 0.02 | 0.02 | 1.18 | 147.00 | 0.240 | 0.16 |
| Substance x Order | -0.10 | 0.02 | -4.18 | 147.00 | **<0.001** | 0.59 |
| Substance x Assessment time | 0.01 | 0.03 | 0.33 | 147.00 | 0.740 | 0.04 |
| Order x Assessment time | -0.03 | 0.02 | -1.39 | 147.00 | 0.168 | 0.19 |
| Substance x Order x Assessment time | 0.06 | 0.03 | 1.84 | 147.00 | 0.067 | 0.26 |
